# Trace element levels: How Substance Use Disorder (SUD) contributes to the alteration of urinary essential and toxic element levels

**DOI:** 10.1371/journal.pone.0294740

**Published:** 2024-02-05

**Authors:** Borhan Mansouri, Nammamali Azadi, Arezo Hashemi Drebadami, Samaneh Nakhaee

**Affiliations:** 1 Substance Abuse Prevention Research Center, Research Institute for Health, Kermanshah University of Medical Sciences, Kermanshah, Iran; 2 Biostatistics Department, School of Public Health, Iran University of Medical Sciences, Tehran, Iran; 3 State Welfare Organization of Kermanshah, Substance Abuse Prevention Research Center, Research Institute for Health, Kermanshah, Iran; 4 Medical Toxicology and Drug Abuse Research Center (MTDRC), Birjand University of Medical Sciences, Birjand, Southern Khorasan, Iran; Hamadan University of Medical Sciences, ISLAMIC REPUBLIC OF IRAN

## Abstract

Increasing illicit drug use is one of the main problems in most countries or societies. Monitoring heavy metals and trace elements in this vulnerable group seems to be necessary. Therefore, we assessed the urinary trace element and toxic metals/metalloids concentrations (Zinc (Zn), Iron (Fe), Copper (Cu), Chromium (Cr), Lead (Pb), Cadmium (Cd), Arsenic (As), Nickel (Ni), and Mercury (Hg)) in opium, tramadol, and cannabis users compared to healthy subjects. In this cross-sectional study, patients with substance use disorder (SUD) (n = 74) were divided into four groups: cannabis, tramadol, opium, and mixed (simultaneous use of more than one of the three studied substances), along with a healthy group (n = 60). Urine samples were prepared by dispersive liquid-liquid microextraction method so that heavy metals/metalloids could be measured by ICP-MS. The mean urinary concentration of Cu (48.15 vs. 25.45; 89.2%, p<0.001), Hg (1.3 vs. 0.10; 1200%, *p* < 0.001), and Zn (301.95 vs. 210; 43.8%, *p* < 0.001) was markedly lower among patients with SUD. The mean urinary concentration of other elements including As (1.9 vs. 4.1; 115.8%), Cd (0.1 vs. 1.10; 1000%), Cr (6.80 vs. 11.65; 71.3%), Ni (2.95 vs. 4.95; 67.8%), and Pb (1.5 vs. 7.9; 426.6%) were significantly higher among patients with SUD compared to healthy subjects. When sub-groups were compared, no significant differences were observed between their trace element levels (Kruskal-Wallis test, *p* > 0.05). This can be an indication that regardless of the type of drug, the levels of trace elements are changed with respect to healthy individuals. Our results showed that illicit drug use causes changes in urinary trace element/heavy metal/metalloid levels and highlights the need for monitoring heavy metals and trace elements in individuals with substance use disorder. Assessment of different elements in biological samples of drug dependents may be useful for implementing new prevention and treatment protocols. In case of changes in their levels, complementary recommendations, attention to diet, and periodic assessment of toxic metal levels within treatment programs will be needed.

## Introduction

Substance use disorder (SUD) refers to the frequent or excessive use of illicit drugs in a way that is harmful to oneself, society, or both [1]. Increasing illicit drug use is one of the main problems in most countries or societies [2]. It directly affects the social and economic features of a country [1]. There are various types of illicit drugs, and most of them may cause malnutrition or nutrient deficiency [1]. Changes in the concentration of trace elements due to substance use have been determined in some previous research [1, 3, 4]. Trace elements play crucial roles in the human body, despite being required in small amounts. They are essential for various biological processes such as energy generation, signal transmission, replication of nucleic acids, transcription and translation, and protection against cellular damage caused by oxidation [5]. Humoral and cellular immune responses are specifically impacted by certain trace elements [6]. Numerous clinical studies have demonstrated a link between the consumption of illicit substances and the occurrence of infectious illnesses. The modification of trace element levels is a potential mechanism that contributes to the impairment of the immune system, thereby elevating the risk of contracting infections [2, 7]. The secretion of extracellular factors that can regulate the activities of immune cells and other cell types involved in the host’s response to infectious agents is affected by the efficiencies of various trace elements. Nevertheless, it is important to maintain a balance between the host’s requirements and the potential harm caused by excessive amounts of redox-active metals like iron and copper, which can cause damage through free radicals. Additionally, infectious agents require the same trace elements for their survival and replication as the host [2, 8]. The activity of certain enzymes that play a role in immune system responses is reliant on trace elements such as Zn and Cu. These enzymes comprise catalase, superoxide dismutase, and glutathione peroxidase [2, 9]. A deficiency in zinc has been shown to result in a specific deficiency in T helper type 1 cell in humans. Increased levels of copper are believed to cause tissue damage by stimulating the production of harmful free radicals. When there is a lack of iron, the activity of protein kinase C (PKC) is reduced in activated T-cells, which affects the movement of PKC within the cell membrane. Iron is also necessary for myeloperoxidase activity, which is involved in the destruction of microorganisms by neutrophils through the creation of highly toxic hydroxyl radicals. During most infections, there is a redistribution of essential trace elements zinc, copper, and iron in the blood serum along with an increase in the production of acute-phase proteins [2]. The lack or overload of any of these trace elements may strongly influence the usual function of human body or cause immunotoxicity [1].

In addition, the heavy metals present in illicit drugs are significant risk factors for toxicity, disability, and mortality in patients with SUD [10]. Heavy metals can accumulate in certain tissues such as blood, kidney, liver, muscle, and bones due to their high chemical stability, low degradation, and bioavailability in the body. This accumulation can lead to various disorders [11]. Over time, heavy metals can become a permanent component of certain body parts such as the bones, kidneys, liver, and brain due to their long half-life. These metals are not biodegradable and remain in the body indefinitely. As they accumulate in higher concentrations, they can form intricate compounds within cells and tissues, which can result in various diseases [12]. Non-essential metals/metalloids such as lead (Pb), cadmium (Cd), and arsenic (As) do not play a role in the normal functions of the body and may even cause harmful results on the health of exposed subjects [13]. Toxicological studies have reported many adverse effects and various symptoms of heavy metals overexposure [10]. Heavy metals have a direct toxic effect on humans, causing various health problems such as immune deficiency, carcinogenicity, cardiovascular disease, cognitive deficits, reproductive and developmental impacts and damage to the nervous system, liver, and kidneys [14, 15]. So, monitoring heavy metals in this vulnerable group seems to be necessary. There are some potential pathways through which substances may be contaminated with heavy metals. Heavy metals may be added to the preparation to increase weight and appreciate its street value. Also, cross-contamination may occur during processing (e.g., during drying). Cannabis/opium is able to uptake heavy metals from substrate soils and deposits these in its tissues due to its bioaccumulative capacity [16, 17].

Illicit drug abuse imposes a large burden on the youth population, and opioid use disorders are still the most common form of drug use disorder in Iran [18]. Due to its geographical location, Iran shares borders with Afghanistan and Pakistan, which has positioned it as a significant conduit for global drug trafficking. This close proximity has facilitated convenient access to drugs and subsequently contributed to a substantial number of drug users within the country [19]. Smugglers combine opium with some heavy metals to gain more weight and profit [17, 20, 21]. Many reports highlighted that opium consumption may alter both toxic and essential element levels [10, 17, 22].

Tramadol use is prevalent nowadays and is associated with toxicity and adverse effects in many cases [20, 23, 24]. Tramadol usage is widespread in Iran, comparable to illegal opioids’ usage despite control measures’ implementation. There have been numerous instances of tramadol abuse, addiction, poisoning, seizures, and hundreds of deaths in recent years [25]. Abuse of this substance can also influence essential trace element absorption such as iron, zinc, copper, and calcium and disrupts the balance of these elements essential for biological activities [26, 27]. There are limited investigations that have evaluated trace element and heavy metal levels in tramadol-dependent individuals [22, 27, 28], which necessitates more research in this field.

*Cannabis* is increasingly being used for recreational, medicinal, and industrial purposes [29, 30]. In recent years there have been reports of increasing trends in cannabis use prevalence in Iran specifically among youths [31, 32]. While cannabis has beneficial properties, it can also cause some concerning health risks. The regular use or abuse of cannabis has been reported to increase the risk of psychosis, respiratory disease, and low birth weight offspring [29]. Cannabis consumers may be exposed to various contaminants such as heavy metals, pesticides, mycotoxins and microbes that can be associated with cannabis products during growing and/or processing stages of plant which requires control and monitoring [29, 30]. Exposure to these contaminants/toxins through cannabis consumption may lead to adverse health effects in short and long term [29].

Although there are some studies assessing serum levels of trace elements and toxic heavy metals in drug dependents; results are conflicting [1, 2, 4, 10, 22]. Also, there is limited previous research on urinary levels of these elements in patients with substance use disorder. Monitoring heavy metals and trace elements in different biological samples seems to be necessary to clarify the issue. Blood and urine are the most useful matrixes for metal bio-monitoring. Urinary and blood levels of trace elements reflected short-term and ongoing exposures respectively and their urinary levels mainly reflect their exposures in last few days [33, 34].

### Aim of the current work

This study aimed to assess urinary trace element and toxic metal concentrations (Zn, Cu, Fe, Cr, Ni, As, Pb, Cd, and Hg) in individuals with opium, tramadol, and cannabis use (some frequently used substances) compared to healthy subjects to create a basis for further research in this field. Based on previous papers, we hypothesized that levels of essential elements may decrease, and some toxic elements may increase in individuals with SUD.

## Materials and methods

### Study characteristics

This cross-sectional study was conducted on patients with SUD in western Iran. Written informed consent was received from all participants. This study protocol was reviewed and approved by the Research and Ethics Committee of Kermanshah University of Medical Sciences (IR.KUMS.REC.1400.398). The authors had no access to information that could identify individual participants during or after data collection. The sample size was estimated using t-test for comparing the mean differences of heavy metal levels between two groups (SUD vs control):

$$n_{1}=\frac{r+1}{r}\times \frac{\sigma^{2}{\left(Z_{1-\beta }+Z_{1-\alpha /2}\right)}^{2}}{{\left(d\right)}^{2}}$$

Where *n*_1_ is the sample size in the SUD group, σ is the common standard deviation, *r* is the ratio of the sample sizes between two groups, and *d* is the least significant difference between the mean concentration levels of two groups. *d*/σ denotes the *Cohen’s d* effect size of the study, α type I error, and 1-*β* the target power of the test. Assuming typical values of 0.05 and 0.80 for the *α* and the power of the test respectively, sample size can be obtained for a pre-defined Cohen effect size value (i.e., 0.50 which is classified as a medium effect size). With these arrangements, the sample size of 62 was enough for each group, assuming both groups were of equal sizes (that is *r* = 1). Sample size was increased to 80 to guard against potential drop-outs. In the end of study, 74 SUD patients and 60 healthy individuals remained in the study. Patients with SUD were divided into four groups: cannabis, tramadol, opium, and mixed (*s*imultaneous use of more than one of three studied substances), along with a healthy group (n = 60) with a total sample size of 134 people. Participants were selected from January to April 2022 from those who referred to Imam Khomeini and Farabi hospitals in Kermanshah. The inclusion criteria for individuals with SUD included people who use drugs in Kermanshah city, and the exclusion criteria included people who had chronic diseases, people with a history of other drug use, people undergoing treatment, cancer or environmental exposure to metal pollutants. The characteristics of the participants including age, gender, BMI, substance consumed, personal and family smoking, and education were recorded using a checklist. After that, 5 ml urine samples were collected and stored at -20°C until analysis for assessment of trace element and toxic metal concentrations (As, Cd, Cr, Cu, Fe, Hg, Ni, Pb, and Zn).

### Sample digestion

Urine samples were prepared by the dispersive liquid-liquid microextraction method so that heavy metals can be measured by ICP-MS. In the dispersive liquid-liquid microextraction method, at first, the sample for extracting metals in urine samples, after preparation, one milliliter of the urine sample was brought to a volume of 5 milliliters with ultrapure water (Milli-Q system, Millipore, Bedford, MA, USA). Then, one milliliter of acetone containing 30 microliters of undecanol (extraction solvent) and 20 microliters of diethyldithiophosphoric acid (complexing ligand) was added to 1 milliliters of urine with a 1.00 milliliter syringe. In this way, a cloudy solution was obtained, and this cloudiness is due to the dispersion of undecanol in water by acetone. Due to the high contact surface of the metal ions with the ligand and extracting solvent, the metal ions immediately form a complex with the DDTP (chelating agent) ligand and are extracted into the undecanol. Next, the solution was centrifuged for 3 min at a speed of 5000 rpm so that the extractant phase (undecanol) floats. The test tube was placed in the freezer for a few minutes to freeze the undecanol, and we transferred it to a clean vial with a small spoon to re-melt at room temperature [35, 36]. Finally, after injecting into the ICP-MS device, the level of the heavy metals under investigation was measured. The validity of the whole procedure was checked against the certified reference material (CRM No. 18). Spikes and control samples were run every six analyses and were adjusted to fit the mid-point of the working range of the methods (recoveries for all elements fell between 94% and 99%). The concentration of heavy metals (As, Cd, Cr, Cu, Fe, Hg, Ni, Pb, and Zn) in this study is in micrograms per liter.

### Statistical analysis

Descriptive results were presented as mean ± SD and number (percentage) as appropriate. Subject characteristics of two groups were compared using Mann–Whitney U tests (Age and BMI) or Chi-squared test (gender, smoking, family smoking, and education). Normality assumption was evaluated using the Shapiro test. Effect sizes were calculated as *r* and Cramer’s *V* for Mann–Whitney U and Chi-squared tests respectively. Furthermore, to compare the concentration values of heavy metals between SUD sub-groups (cannabis, opium, tramadol, and multi-drug abusers), Kruskal–Wallis test with *epsilon*-squared as effect sizes was used. Spearman’s rank correlations were employed to measure the association between trace elements in two groups. In addition, rank-based robust regression analysis was performed to evaluate the effect of multifactorial covariates including group, gender, and age on metal concentration levels. All statistical analyses were performed using R Statistical Software (version 4.2.1; R Foundation for Statistical Computing, Vienna, Austria).

## Results

### Demographic characteristics

Table 1 reports the characteristics of the participants organized into two groups: SUD (n = 74, 55%) and healthy individuals (n = 60, 45%). The two groups were comparable in terms of age and gender. Individuals with SUD were mostly cigarette smokers (81.1% vs. 13.3% in the healthy group), had lower education levels (43% with education less than a high school degree vs. 23% in the healthy group), and also had significantly lower weight (mean BMI = 23.64 vs. 26.60 in the healthy group, *p* < 0.001, effect size r = -0.471 indicating a large difference).

**Table 1:** Characteristics of participants in the study.

Participant characteristics	SUD group (n = 74)	healthy group (n = 60)	Total (n = 134)	P-value
**Age**	34.28 ± 9.65	34.28 ± 8.90	34.28 ± 9.28	0.934*
**BMI**	23.64 ± 3.90	26.60 ± 3.37	24.97 ± 3.95	**< 0.001** *
**Gender**				
Male	34 (45.95%)	33 (55%)	67 (50%)	0.385^†^
Female	40 (54.05%)	27 (45%)	67 (50%)	
**Family smoking**				
Yes	27 (36.49%)	11 (18.33%)	38 (28.36%)	**0.033** ^†^
No	47 (63.51%)	49 (81.67%)	96 (71.64%)	
**Smoking**				
Yes	60 (81.08%)	8 (13.33%)	68 (50.75%)	**< 0.001** ^†^
No	14 (18.92%)	52 (86.67%)	66 (49.25%)	
**Education**				
< high school	32 (43.24%)	14 (23.33%)	46 (34.33%)	**0.028** ^†^
High school	27 (36.49%)	24 (40%)	51 (38.06%)	
Academic degree	15 (20.27%)	22 (36.67%)	37 (27.61%)	

* The p-value was calculated using Mann-Whitney U test.

† The p-value was obtained using chi-squared test. SUD: substance use disorder

Table 2 reports the levels of trace elements (μg L^-1^) in median and quartile ranges for both studied groups. Compared to the SUD group, the levels of Cu (48.15 vs. 25.45 μg L^-1^; 89.2% increase, *p* < 0.001), Hg (1.3 vs. 0.10 μg L^-1^; 1200% increase, *p* < 0.001), and Zn (301.95 vs. 210 μg L^-1^; 43.8% increase, *p* < 0.001) were markedly higher among healthy individuals. The mean urinary concentration of As (1.9 vs. 4.1 μg L^-1^; 115.8% increase), Cd (0.1 vs. 1.10 μg L^-1^; 1000% increase), Cr (6.80 vs. 11.65 μg L^-1^; 71.3% increase), Ni (2.95 vs. 4.95 μg L^-1^; 67.8% increase), and Pb (1.5 vs. 7.9 μg L^-1^; 426.6% increase) were significantly lower among the healthy group compared to SUDs. Since all trace elements except for Fe showed noticeable level changes between the two studied groups, it is worthwhile to see if such level discrepancies can be seen between SUD sub-groups.

**Table 2:** The urinary levels of essential and toxic elements (μg L^-1^) among both patients with SUD and healthy individuals are presented as median (25th– 75th percentile).

Element	SUD group	Healthy group	Total	*p*-value
As	4.1 (2.9–5.57)	1.9 (0.2–3.50)	3.3 (1.92–5.05)	**< 0.001**
Cd	1.1 (0.52–2.17)	0.1 (0.1–0.2)	0.5 (0.1–1.1)	**< 0.001**
Cr	11.65 (8.77–15.35)	6.80 (4.80–11.50)	10.2 (6.05–14.27)	**< 0.001**
Cu	25.45 (15.27–37.02)	48.15 (35.35–70.17)	36.0 (22.27–52.9)	**< 0.001**
Fe	160.10 (131.10–594.60)	349.25 (241.42–433.95)	310.3 (141.55–489.62)	**0.05002**
Hg	0.1 (0.1–1.47)	1.3 (1.2–1.40)	1.1 (0.1–1.4)	**< 0.001**
Ni	4.95 (2.7–12.0)	2.95 (2.5–3.75)	3.25 (2.52–10.85)	**< 0.001**
Pb	7.9 (4.95–13.77)	1.5 (0.77–3.02)	4.7 (1.75–8.57)	**< 0.001**
Zn	210.0 (160.0–367.5)	301.95 (190.1–387.6)	250.16 (166.82–385.52)	**0.044**

SUD: substance use disorder

Table 3 summarizes the urinary concentrations of trace elements in drug-user sub-groups. Interestingly, when the sub-groups were compared, no significant differences were observed between their trace element levels (Kruskal-Wallis test, *p* > 0.05).

**Table 3:** The urinary levels of essential and toxic elements (μg L^-1^) among patients with SUD (Opium, cannabis, tramadol, and mixed drug) are presented as median (25th– 75th percentile).

Element	Cannabis	Opium	Tramadol	Mixed	*p*-value
As	4.45 (3.32–6.10)	3.70 (2.40–4.70)	4.15 (3.35–4.72)	4.30 (3.25–8.65)	0.317
Cd	1.1 (0.80–2.25)	0.9 (0.30–2.10)	1.1 (0.80–1.95)	1.1 (0.55–2.20)	0.575
Cr	10.25 (8.77–13.87)	13.20 (10.0–16.30)	13.80 (12.95–14.97)	10.90 (7.65–15.0)	0.337
Cu	23.1 (15.82–33.37)	26.9 (16.20–37.70)	36.3 (25.80–47.57)	19.2 (14.05–41.40)	0.520
Hg	0.10 (0.1–1.32)	0.10 (0.1–0.75)	0.25 (0.1–0.75)	0.1 (0.1–6.80)	0.397
Fe	149.0 (124.6–424.0)	160.1 (134.2–594.3)	151.2 (132.8–211.8)	526.9 (149.0–661.2)	0.232
Ni	3.50 (2.55–11.87)	3.20 (2.40–12.0)	7.95 (3.62–12.67)	11.10 (2.40–12.0)	0.294
Pb	8.85 (4.75–14.02)	6.90 (5.30–10.60)	8.0 (7.25–11.52)	8.0 (5.20–14.3)	0.901
Zn	205 (142.5–250.0)	190 (130–390)	375 (187.5–437.5)	290 (180–390)	0.070

To explore whether the levels of trace elements vary with smoking habit, duration of drug use, education, and BMI of individuals, a robust regression model was performed. Table 4 reports the coefficients of this model. Element Hg was removed from the analysis due to a large number of tie values (over 50% of data). As expected, the differences in the levels of almost all trace elements between the two studied groups were also confirmed by the robust regression model.

**Table 4:** The coefficient estimates of the effect of covariates under robust regression analysis.

	Trace element							
Covariate	As	Cd	Cr	Cu	Fe	Ni	Pb	Zn
BMI	-0.002	0.001	-0.334**	0.112	9.283*	0.097	0.180*	-2.229
Usage Duration	-0.004	-0.001	-0.006	0.027	0.418	0.016	-0.003	0.728*
Smoking (No)	0.855	-0.265	-0.099	0.389	24.95	1.376	-0.137	26.05
Family Smoking (N)	-0.807	0.165	-1.072	-2.392	58.89	-0.435	0.160	-6.224
Group (Healthy)	2.40***	1.023***	3.669**	-23.22***	-79.61	1.704	6.843***	-78.61*
Education								
High School	0.157	-0.093	0.170	-0.048	-4.319	-1.445	0.128	14.00
Academic	1. 21**	0.006	0.672	-0.017	-8.916	0.201	1.247	21.25

* Significant at 0.05 level

** significant at 0.01 level

*** highly significant at 0.001 level

Body mass index significantly affected the levels of Cr (β = -0.334, p = 0.004), Fe (β = 9.283, p = 0.032), and Pb (β = 0.180, p = 0.024), so that for every one-unit increase in the BMI variable, Cr levels decreased by 0.334, but Fe and Pb levels increased by 9.28 and 0.180, respectively. Duration of drug use increased the Zn levels but had no effect on the concentration levels of other elements. Having an academic degree increased the levels of As by 1.21 compared to those with less than a high school education.

### Correlation analysis

**Fig 1:**
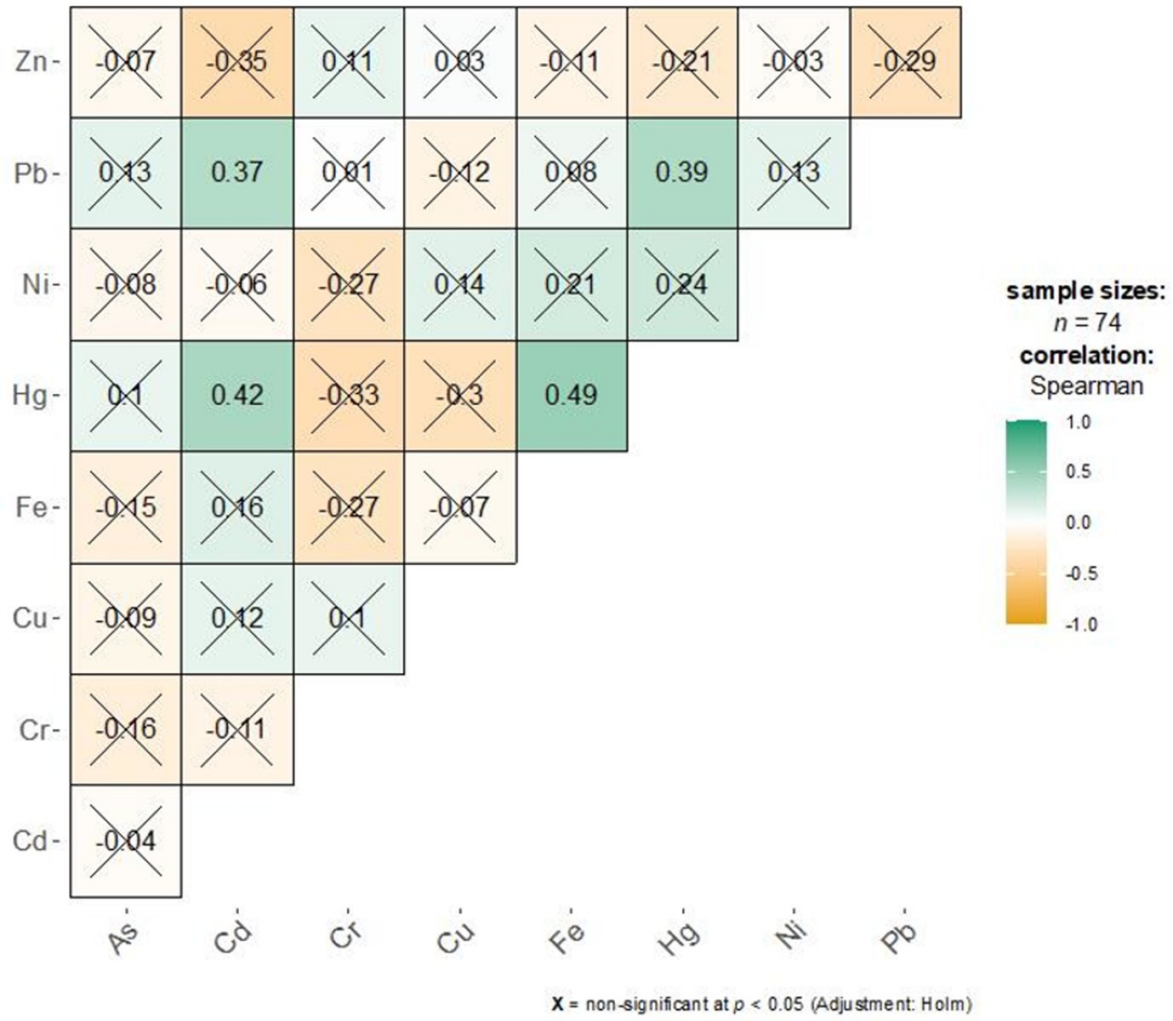
Correlation between trace element pairs for the SUD group.

**Fig 2:**
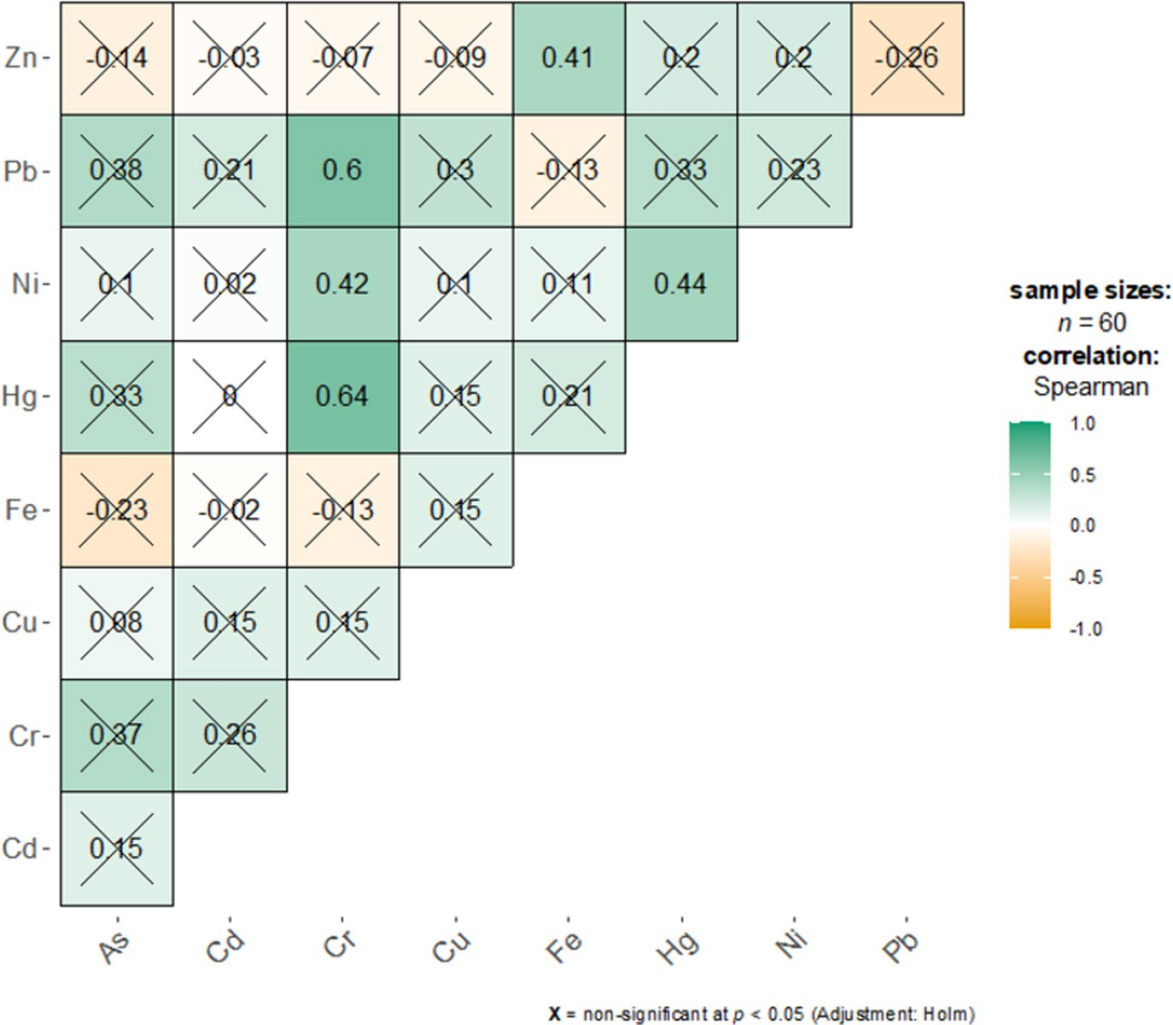
Correlation between trace element pairs for the healthy group.

Figs 1 and 2 show the Spearman correlation between trace elements for the drug-dependent and healthy groups, respectively. Due to the non-normal nature of the data, Spearman correlation was used to assess the association between pairs. Pairs of significant correlations at the nominal 5% level were specified in green for positive or orange for negative correlations. A cross sign (X) was used to highlight non-significant correlations. For the SUD group, most pairs were non-significant. Moderate correlations were observed between Pb-Cd (r = 0.37, p = 0.035), Pb-Hg (r = 0.39, p = 0.022), Hg-Cd (r = 0.42, p = 0.006), and Hg-Fe (r = 0.49, p = 0.0002) pairs. For the healthy group, most correlations were positive and non-significant except for Cr-Ni (r = 0.42, p = 0.003), Cr-Pb (r = 0.60, p<0.001), Cr-Hg (r = 0.64, p<0.001), Hg-Ni (r = 0.44, p = 0.002), and Zn-Fe (r = 0.41, p = 0.003) pairs.

## Discussion

SUD can lead to a series of adverse outcomes at psychological, social, and emotional levels. In addition, it can greatly affect the dietary habits and nutritional status of individuals [37]. The intake of the majority of minerals and vitamins is below the recommended values in this population [37], and different types of illicit drugs may cause malnutrition or nutrient deficiency, which are probable causes of immune deficiency in patients with SUD [1]. In the current study, we found lower urinary levels of zinc and copper in the group of patients with SUD compared to the healthy group. There was no significant difference in the level of iron between addicted and healthy individuals. The lower urinary levels of trace elements may reflect their lower levels in the serum of patients with SUD. Various factors may be responsible for changing the levels of essential trace elements. Many studies of this population have shown that the nutritional status of persons with SUD can be changed. They may have deficient feeding and nutrient intake [38–42]. The side effects of illicit drugs, including anorexia, constipation, abdominal pain, and vomiting, can affect the function of the gastrointestinal tract and induce various mineral deficiencies in the body as well as malnutrition [37, 43]. Also, the variations in essential trace element concentrations may be due to smoking habits, inflammation, and peripheral responses of the body to increased oxidative stress due to SUD [37, 44].

Previous studies have assessed trace elements in different biological samples and various types of illicit drugs. For example, Akbari (2015) showed that serum copper was higher in opium, heroin, and methamphetamine users compared to the healthy group, and no significant difference in serum zinc levels was reported. Also, serum iron levels were lower in methamphetamine and heroin users compared to healthy individuals [2]. El-Safty et al. (2018) did not report a significant difference in urinary copper elimination in tramadol users (n = 18) compared to non-users (n = 19), and tramadol use could not influence nephron integrity for reabsorption of copper [27]. Martinez et al. investigated some trace element levels (Zn, Cu, Mg, Fe, and lithium) in the plasma and erythrocytes of parenteral patients with SUD (69 heroin and 31 heroin with other drugs) and controls (n = 20). Plasma Zn and Fe levels in erythrocytes decreased, while Cu levels in plasma and erythrocytes increased significantly in the group of patients with SUD. On the other hand, plasma Zn levels were higher in participants with a longer abstinence period than those who took the drugs before the study, indicating that the drugs have acute and reversible effects on plasma Zn levels [45]. In their study, plasma iron levels in addicted people did not show a significant difference compared to healthy people [45]. In another study, the serum concentrations of trace elements such as zinc, copper, iron, and magnesium were investigated in patients with SUD (most of them used ganja, heroin, and phensidyl). Imbalances in the levels of micronutrients, immunoglobulin, and antioxidants were observed in substance abuse patients, which tend to return to control values after detoxification. They reported that patients with SUD had higher serum copper levels and lower levels of iron compared to the control group [1].

The possible reasons for discrepancies in the results of previous literature may be due to differences in the duration and type of drug use, differences in biological samples, environmental conditions, genetic or racial factors, and variations in the methods used for analysis.

In addition to the effects of opioids on essential trace element levels, some investigations have specified the effects of these elements on opioids’ pharmacodynamics. For example, one research showed that zinc deficiency may exacerbate opioid use. They reported that patients with zinc deficiency should multiply the amount of opioid used to reach analgesia similar to patients with normal zinc status, and systemic administration of zinc increased the analgesic effects of tramadol [46]. Another study reported that zinc, magnesium, and manganese administration increased tramadol-induced analgesia assessed using hot-plate and tail-flick tests in animals [47]. Although the exact way in which deficiencies of essential trace elements contribute to opioid addiction and tolerance is not yet clear, there are several proposed theories. For example, one theory suggests that Zn interacts with specific sulfhydryl (SH) groups within the opioid receptor’s biochemical structure, causing a decrease in receptor affinity and the number of sites available for binding to exogenous opioids. Zn is believed to oxidize these SH groups through a reversible redox reaction, leading to the prevention of opioids binding to the receptor. Additionally, Zn is involved in regulating MOR activation of the protein kinase C gamma (PKCγ) cascade, which plays a role in the development of tolerance and addiction [46]. The integration of the above-mentioned conditions can present the interactions of SUD and trace elements as follows: SUD may result in trace element alteration in the body, and this situation may increase the need for substance use, thereby forming a vicious cycle. In this study, we also found elevated urinary levels of some toxic elements (As, Cd, Cr, Ni, Hg, and Pb) in the group of patients with SUD compared to healthy individuals. The presence of heavy metals in illicit substances is a major risk factor for disability and premature loss of life for individuals with SUD. Their association with some adverse effects including damage to the nervous system, liver, and kidneys; anemia; hypertension; cardiovascular disease; carcinogenic effects; immune deficiency; infertility; developmental problems including cognitive deficits, learning disability; and memory loss has been reported [12, 48]. Substances can become contaminated with heavy metals through various pathways, such as adding heavy metals to increase weight and value, cross-contamination during processing, and uptake from substrate soils [16, 17]. A high tendency for opium poppy and cannabis to accumulate toxic heavy metals, especially cadmium and lead, chromium, as well as semi-metal arsenic has been documented previously [16, 49–55].

Previous studies have reported elevated concentrations of heavy metals in opium users in different biological samples [10, 56–60]. Similar to our findings, Azadi et al. (2022) determined elevated urinary concentrations of Cd, Pb, Cr, and Co in opium-dependent subjects compared to controls [10]. Many other studies have reported elevated blood concentrations of heavy metals in opium users [56–60]. In interpreting past studies, some factors such as nutritional status of opium addicts, route of opium use, method and length of opium consumption should be considered since they can affect the accumulation of toxic metals in the body [61]. Alongside opium-induced metal accumulation in the body, some researchers have demonstrated the effects of these toxic metals on the pharmacodynamics of opioids [62]. For example, it has been suggested that lead may have disruptive effects on neural pathways related to the development of opioid addiction. It can change dopamine metabolism and the expression of dopamine receptors, and it can also increase morphine tolerance [62]. The antagonistic effects of Cd on mu-opioid receptors and inhibitory effects on dopamine release have been suggested [62–64]. Decreased response to morphine due to cadmium exposure has been reported in some experimental investigations [63].

In line with our results, Farnia et al. (2022) showed significantly higher urinary concentrations of Pb, Cr, Cd, and Ni in tramadol-dependent individuals [22]. To the best of our knowledge, there are limited studies that have evaluated most of these metals in tramadol users, which warrants further study in this area.

There are case reports of lead and arsenic poisoning due to cannabis use [16, 65]. For example, there is a case series of 95 patients with lead toxicity due to Pb contamination of cannabis to increase its weight [65]. Also, some documents reveal high levels of Pb and As in the cannabis samples [66, 67], which are more than the permissible amount for medicinal plants determined by the World Health Organization [67]. Based on another finding of the current study, there were no significant differences in trace element levels in different sub-groups of drug users. This can be an indication that regardless of the type of drug, the levels of trace elements are changed with respect to healthy persons. It highlights the need for toxic or essential trace element monitoring in all studied drug types for preventive measurements in patients with SUD.

Finally, it should be considered that the effects of an increase/decrease in the concentration of a single essential or toxic element are not limited to this element alone. Still, the whole distribution pattern of the elements in the biological system will be affected. Therefore, interdependencies and interactions of certain elements with each other seem to be crucial [1].

## Conclusion

Our results showed that illicit drug use induces changes in urine essential element/heavy metal levels. Patients with SUD had lower levels of some trace elements (Zn, Cu, and Hg) and significantly elevated levels of some toxic heavy metals (Pb, As, Cd, Cr, and Ni). Identification of different elements in biological samples of drug dependents may be useful for implementing new prevention/treatment protocols. In the case of changes in their levels, complementary recommendations, attention to diet, and periodic assessment of toxic metal levels within treatment programs will be needed.

A limitation of the current study was that complete information on the dietary habits of the participants or the effects of dietary intake on trace element status and environmental factors was not applicable. Therefore, it is not clear whether the changes are due to the effect of drug use or nutritional status of opioid users or environmental conditions. In addition, cytokine assays were not performed, which can be considered in future studies. For example, evaluating how drug dependence affects toxic and essential trace element concentrations taking into account the environmental factors and nutritional status of participants by using large-scale research in different samples such as urine, nail, and hair can be considered a field of future studies. This study should be continued to clarify the potential mechanisms of trace element alterations during exposure to illegal drugs.

## Supporting information

S1 Checklist *PLOS ONE* clinical studies checklist. (DOCX)
